# Peoples expectations from healthcare providers – a Turkish perspective

**DOI:** 10.1186/1472-6963-12-S1-P7

**Published:** 2012-11-21

**Authors:** Saad A Ali Jadoo Alazawi, Syed Aljunid, Seher Nur Sulkus, Zafar Ahmed, Sharifa Ezat WP

**Affiliations:** 1 United Nation University International Institute for Global Health, Kuala Lumpur, Malaysia; 2 Department of Econometrics, Economics and Management Sciences Faculty, Gazi University, Ankara, Turkey; 3 International Centre for Casemix and Clinical Coding, UKMMC,Kuala Lumpur, Malaysia; 4 Department of Community Health, Faculty of Medicine, National University of Malaysia, Kuala Lumpur, Malaysia

## Background

There is high expectation from the population on part of the healthcare providers. These include; skillful and timely medication administration; and knowledge, honesty, listening skills, availability and professional attitude. The aim of this paper is to evaluate the expectation of population with regards to the healthcare providers in Turkey.

## Methodology

A cross sectional study in Turkey, including both rural and urban population, carried out from October 2011 till January 2012. A total of 540 heads of household were selected using multi stage random sampling. Data was collected using structured self-administered questionnaire. The tools used was modified 16-item Quality of Care Through the Patients’ Eyes (QUOTE) questionnaire. QUOTE questionnaire measures communication/accessibility, organizational skills and professional skills. The response rate was (77.1%) and data was analyzed by using SPSS version 16.0.

## Results

All aspects measured using QUOTE questionnaire were found to be important or extremely important by the respondent, but with varying degrees of priority. Quality aspects related to the professional skills of physicians came first followed by communication or accessibility and last but not the least are the organizational skills of health care providers.

## Conclusion

This study explored the Turkish people priorities and expectations regarding healthcare providers. Level of expectation varies across the population. This may reflect the need to understand people’s expectations before providing the services to avoid complaints that may occur after the services have been rendered.

